# A Rare Case of Endolymphatic Sac Tumour: Clinicopathologic Study and Surgical Management

**DOI:** 10.1155/2014/376761

**Published:** 2014-06-04

**Authors:** Emanuele Ferri, Maurizio Amadori, Enrico Armato, Ida Pavon

**Affiliations:** ^1^Emergency Department, Otorhinolaryngology Unit, Otosurgery and Audiovestibology Section, General Hospital of Monselice, Via G. Marconi 19, Padua, 35043 Monselice, Italy; ^2^Surgical Department, Otorhinolaryngology Unit, Otosurgery, Audiology and Vestibology Section, General Hospitals of Dolo and Mirano, Via Mariutto 76, Venice, 30035 Mirano, Italy; ^3^Surgical Pathology Unit, General Hospitals of Dolo and Mirano, Via Mariutto 76, Venice, 30035 Mirano, Italy

## Abstract

*Objective*. Endolymphatic sac tumor (ELST) is a rare neoplasm arising from the intrapetrous portion of the endolymphatic sac, either isolated or in association with the von Hippel-Lindau disease. We report a sporadic case of ELST with an overview of the literature and a discussion of clinic-radiological, histopathologic, and surgical findings. *Case Report*. A young woman presented with a progressive hearing loss in the left ear. Otoscopy showed a reddish, bleeding hypotympanic mass. CT demonstrated an expansile lytic mastoid lesion extending to the middle ear, with bone erosion. MRI confirmed a lesion of increased signal on T1-weighted sequences. The patient underwent a canal wall-down tympanoplasty with complete removal of the tumor. Histopathology was consistent with a papillary ELST. Immunohistochemistry was positive for cytokeratin and chromogranin A. *Conclusion*. This paper highlights the rarity of ELST, the need for an accurate neuroradiological and immunohistochemical study at the early stages, and the timeliness of surgical treatment.

## 1. Introduction

Endolymphatic sac tumors (ELSTs) are very rare nonmetastasizing neuroectodermal tumors arising from the proximal, intrapetrous portion of the endolymphatic sac. The first report concerning the ELST was in 1984 [1], but it is in 1989 that Heffner characterized ELST as a tumor deriving from the endolymphatic sac epithelium of the internal ear, referring to it as a “low-grade adenocarcinoma” [2].

Since this time, a number of articles have reported on these tumors, along with their association with the von Hippel-Lindau (VHL) disease [3–7]. The reason for this increase in reporting is most likely attributable to an increased awareness that these lesions, which were previously categorized as aggressive papillary middle ear tumors, are indeed a distinct pathologic entity arising from the endolymphatic sac. The detection of immunohistochemical markers has aided significantly in this distinction [3]. ELSTs usually occur in isolation, although some arise in association with the von Hippel-Lindau disease (VHL), a rare genetic disorder caused by a mutation of the chromosome 3 (3p25-26) [8]. The incidence of ELST in the adult population is 1 : 30,000 and in the VHL population is around 10% [9].

We report a sporadic case of a young woman with an ELST not vHL related, underlining the difficult differential diagnosis and emphasizing the role of immunohistochemical study and the surgery as the treatment of choice.

## 2. Case Report

**Figure 1:**
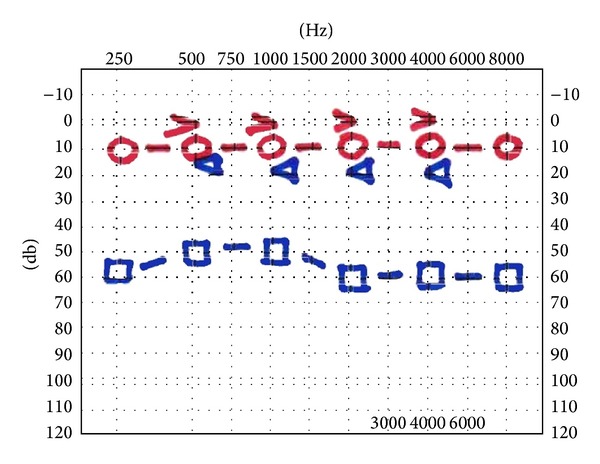
A pure-tone audiometry before surgical treatment shows a moderate, pantonal, conductive hearing loss in the left ear.

**Figure 2:**
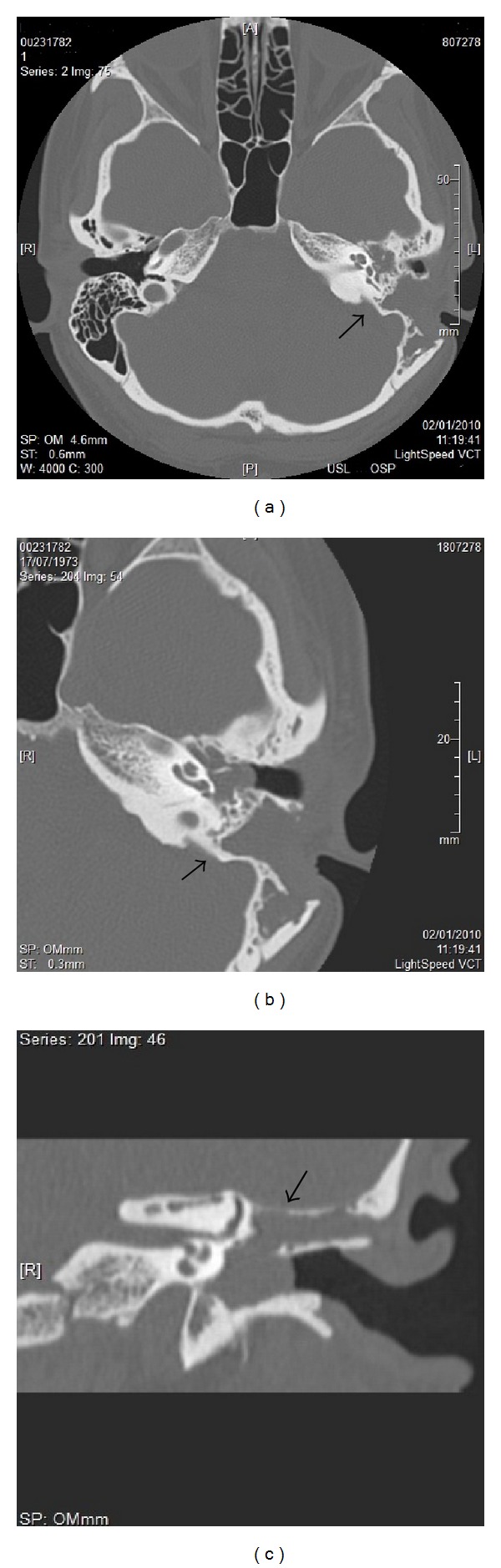
CT imaging (a) with axial (b) and coronal (c) scans demonstrated an irregular, hypodense, and expansile lytic lesion of the mastoid process of the left petrous bone extending to involve both the left medial mastoid and the middle ear, with a subtotal erosion of ossicular chain and a partial bone destruction of the “tegmen tympani” (see arrows). The mass did not appear to affect the semicircular canals, the canal of the facial nerve, and the internal auditory canal.

**Figure 3:**
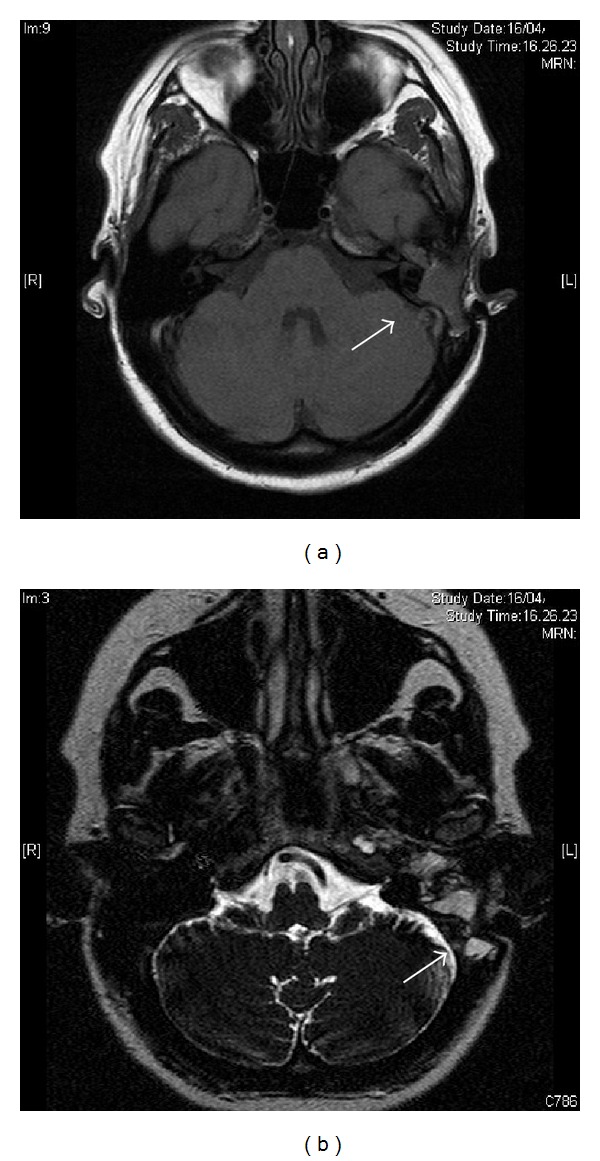
Axial T1-weighted cerebral MRI confirmed the presence of a lesion of increased signal, similar to brain tissue (a); the tumor (22 × 15 × 11 mm) enhanced with gadolinium in the medial portion of middle ear and in the tubaric region (b).

**Figure 4:**
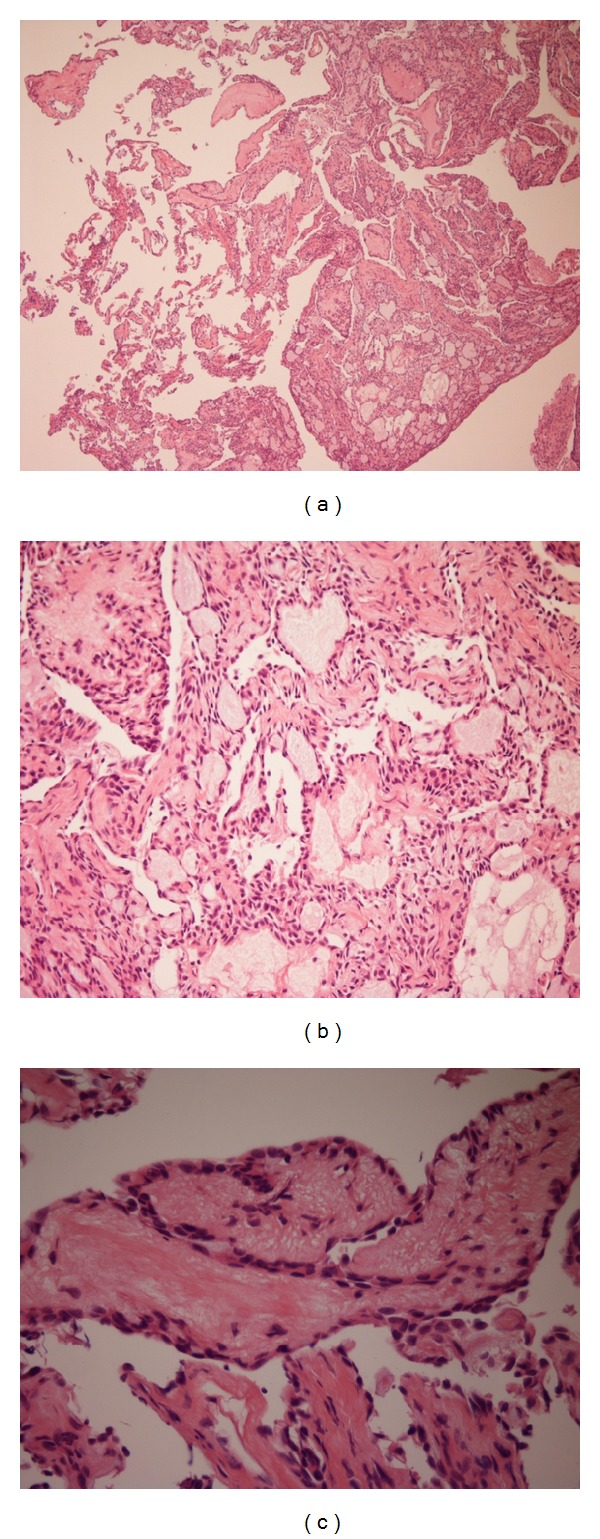
Histopathologic study of ELST. The histologic sections showed the papillary and glandular-cystic architecture of the ELST (a) (H&E, 50x). There were cystic glandular spaces filled with colloid-like material which was remarkably similar to thyroid tissue (H&E, 200x) (b). The papillary structures were lined by a single layer of low cuboidal epithelial cells. There were minimal cellular pleomorphism and rare mitotic activity (H&E, 200x) (c).

**Figure 5:**
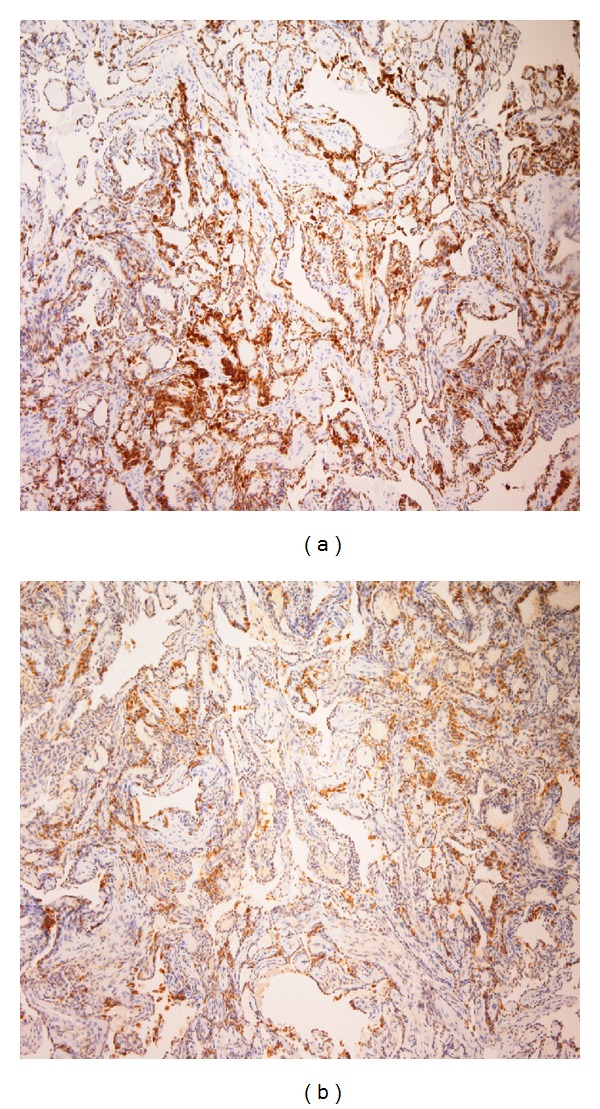
Immunohistochemical study of ELST. The tumor cells showed positive reactivity with pan-cytokeratin MNF116 (a) and chromogranin A (b) (immunostaining, 100x).

**Figure 6:**
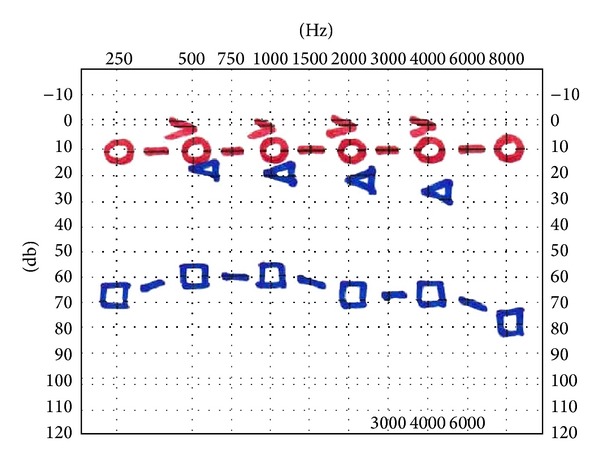
At 36-month follow-up a pure-tone audiometry identifies any worsening of hearing loss.

A 37-year-old woman presented with a 3-month history of fullness and progressive hearing loss in the left ear. There was no tinnitus, otalgia, otorrhea, vertigo, or facial nerve paralysis. Otomicroscopy of the left ear showed the presence of a reddish, easily bleeding hypotympanic mass, extending to the medial third of the external auditory canal. Examination of the facial nerves, nasopharynx, oral cavity, larynx, and neck was normal. Neither the symptoms nor a family history of VHL disease was found in the patient. The pure-tone audiometry revealed a moderate, pantonal, conductive hearing loss in the left ear (Figure 1). Computed tomography (CT) demonstrated an irregular, hypodense, and expansile lytic lesion of the mastoid process of the left petrous bone extending to the middle ear, with a subtotal erosion of ossicular chain and the “tegmen tympani.” The mass did not appear to affect the semicircular canals, the canal of the facial nerve, and the internal auditory canal (Figure 2). Cerebral magnetic resonance (MR) confirmed the presence of a lesion of increased signal on T1-weighted sequences, enhanced in the medial portion of the middle ear and in the tubaric region (Figure 3). The patient underwent a canal wall-down tympanoplasty and complete removal of the tumor. The facial nerve was exposed to a tract of about 3 mm at the level of the second genu. The postoperative follow-up was uneventful. Histopathology was consistent with ELST. The tumor presented the architecture of a papillary adenocarcinoma of low histologic grade. There was no evidence of mitosis or necrosis (Figure 4). Immunohistochemical studies were positive for CK MNF116 (cytokeratin wide spectrum—types 5, 6, 8, 17, and 19) and chromogranin A (Figure 5), with a Ki67 not being significant. The tumor did not stain with GFAP (glial fibrillar acid protein), synaptophysin, S100, calponin, vimentin, and thyroglobulin. Genetic testing for the von Hippel-Lindau disease (mutation of chromosome 3p25/26) was negative. A postoperative MRI revealed no residual tumor. Radiological and clinical evaluations at 36-month follow-up demonstrated no evidence of recurrence and a pure-tone audiometry identified any worsening of hearing loss (Figure 6).

## 3. Discussion

ELST is a primary neoplasm of the temporal bone; till now, less than 100 cases have been reported in literature. Most of them are sporadic, but 30% are associated with the VHL disease [10–22]. Mean age of patients with sporadic cases is 52 years, whereas in patients with VHL disease it is 31 years; female-to-male ratio is 2 : 1 in VHL disease patients and 1 : 1 in non-VHL disease patients [11]. Patients characteristically present with unilateral sensorineural hearing loss, tinnitus, otalgia, otorrhea, vertigo, ataxia, and facial nerve paresis. At the time of diagnosis, clinical audiovestibular features have usually been present for many years. As the symptoms may mimic those of Ménière's disease, radiologic imaging and histopathology are essential for the diagnosis.

The endolymphatic sac is situated between the petrous periosteum and the dura mater of the posterior cranial fossa, posterior to the internal auditory canal. Tumors growing there slowly infiltrate and erode the petrous bone. Bambakidis et al. propose a grading system for ELSTs based on radiographic location and extent [11]. In practice, however, it is hard to correlate a patient's prognosis with the tumor's grade using their classification system. Surgery with complete resection at an early stage leads to a long disease-free interval. Metastases from ELST are very rare.

CT of these tumors is characterized by bony erosion centered on the posteromedial aspect of the petrous bone, although small tumors may show bony abnormalities other than widening of the vestibular aqueduct. Most cases demonstrate a thin calcified peripheral rim representing expanded cortex of the petrous bone. CT can be used to demonstrate normal architecture of the jugular foramen, thus, differentiating the mass from a jugular glomus or other jugulotympanic mass. In addition, CT will show typical sparing of the cochlea and middle ear [7, 23].

On MR imaging, the ELSTs usually have a heterogeneous appearance. Multiple high-signal intensity foci on both T1- and T2-weighted images indicate the presence of blood-, methemoglobin-, or proteinaceous-filled cysts or cholesterol clefts. Such blood products are rare in other temporal bone lesions. Scattered low-signal foci represent hemosiderin deposition due to chronic parenchymal bleeding. Calcifications and vessels can demonstrate signal voids. The addition of contrast results in a nonhomogenous enhancement of the tumor's solid portions [23].

ELSTs are papillary adenocarcinomas of low histologic grade. In the past, they have been confused with middle ear carcinomas and ceruminous gland tumors. Initial difficulties in the differentiation of ELSTs from choroid plexus papilloma were aided by the recognition of transthyretin as a site-specific marker for choroid plexus epithelium [6, 24]. Many antigens are expressed by ELST and are detectable with immunohistochemical analysis. ELST typically stains positive for cytokeratins, epithelial membrane antigen (EMA), vimentin, and periodic acid-Schiff, while detection of S100 protein and/or neural-related antigens such as synaptophysin and glial fibrillar acid protein (GFAP) is variable. ELST does not show reactivity for calponin and calretinin [3, 5] (Table 1). The tumors must be distinguished histologically from metastatic clear cell carcinoma of renal origin, which is also highly vascular but shows nuclear atypia and is more commonly tubular than papillary. Thyroid-like areas in ELSTs can also suggest metastatic follicular thyroid carcinoma. In the latter, thyroglobulin stains will be negative and clear cell papillary areas are not seen [5].

In this case report the positivity for chromogranin A is a rare but interesting remark. The reactivity of the epithelial cells with antibodies against neuron specific enolase, chromogranin, and somatostatin, respectively, demonstrates a paracrine activity of the endolymphatic sac [25]. In 1995 Bold et al. postulate that temporal bone adenomas have neuroendocrine characteristics, are positive for neuroectodermal markers (including chromogranin), and could be derived from the specialized neuroectoderm of the neural crest [26]. Recently, Duderstadt et al. confirm the importance of immunohistochemistry but reveal that a differentiation between the benign middle ear adenoma and aggressive papillary tumour is very hard [27].

The differential diagnosis of ELSTs includes other deep temporal bone lesions. Jugular glomus and jugulotympanic paragangliomas involve the jugular foramen and then the middle ear rather than retrolabyrinthine temporal bone. Irregular bone destruction by paragangliomas is similar to ELSTs, as are flow voids, marked enhancement, and a predominant external carotid artery supply. Bone spicules in the tumor matrix and high-signal hemorrhagic foci in paragangliomas are unusual. Hypervascular metastases such as renal cell or papillary thyroid carcinoma may also produce a destructive deep temporal bone lesion. They lack internal hemorrhagic foci on MRI examination. Schwannomas of the jugular foramen are well defined and centered in the jugular foramen, not in a retrolabyrinthine location. In the case of petrous apex granuloma, high signal is present on unenhanced T1-weighted MRI examination, but petrous apex granuloma is distinguished from ELSTs by its characteristic location [28].

Concerning treatment, radical surgical local excision remains the mainstay of current therapy. In our case the timeliness of surgical treatment with a canal-down tympanoplasty allowed a complete removal of the lesion with a good long-term follow-up. The surgery may sometimes necessitate sacrifice of cranial nerves, and sometimes total resection of the advanced tumors may be impossible due to the anatomic complexity [6, 29], so postoperative radiotherapy was suggested as adjuvant therapy in most cases. In addition, radiotherapy may also be suitable as a salvage treatment in recurrent ELSTs [18, 30]. In our case, the complete removal of the tumor was possible because of the small size of the mass that did not need preoperative embolization. Other treatment options such as gamma or cyber-knife need to be evaluated on larger series [21].

The ELST shows quite a large variation in tumor extension (grade), hearing level, and comorbidity. Consequently, in our opinion, treatment must be individualized. Although surgery might be attractive as the first treatment option, radiotherapy and wait-and-scan are an alternative especially when hearing is still intact.

## Figures and Tables

**Table 1:** Immunohistochemical staining profile of ELST.

Immunohistochemical staining profile of ELST	
Cytokeratins (types 5, 6, 8, 17, and 19)	+
Epithelial membrane antigen (EMA)	+
Vimentin	+
Periodic acid-Schiff	+
S100 protein	+/−
Glial fibrillar acid protein (GFAP)	+/−
Chromogranin A	+/−
Synaptophysin	+/−
Neuron specific enolase (NSE)	+/−
Thyroglobulin	−
Calponin	−
Transthyretin	−
Calretinin	−
